# IL-15-dependent balance between Foxp3 and RORγt expression impacts inflammatory bowel disease

**DOI:** 10.1038/ncomms10888

**Published:** 2016-03-11

**Authors:** Milena J. Tosiek, Laurence Fiette, Sary El Daker, Gérard Eberl, Antonio A. Freitas

**Affiliations:** 1 Unité de Biologie des Populations Lymphocytaires, Department of Immunology, Institut Pasteur, 25, rue du Docteur Roux, 75015 Paris, France; 2 CNRS, URA1961, 75015 Paris, France; 3 Unité d'Histopathologie Humaine et Modèles Animaux, Department of Infection and Epidemiology, Institut Pasteur, Hôpital Ste Anne, 75015 Paris, France; 4 Université Paris-Descartes, Hôpital Ste Anne, 75015 Paris, France; 5 Unité de Développement des Tissus Lymphoïdes, Department of Immunology, Institut Pasteur, Paris, France

## Abstract

The ability of CD4+ T cells to change their phenotype and to specialize into different functional subsets may enhance the risk of autoimmune diseases. Here we investigate how a pleiotropic cytokine interleukin (IL)-15 may modify the functional commitment of CD4+ T cells expressing the lineage-associated transcription factors: forkhead box P3 (Foxp3; Treg) and RORγt (Th17) in the context of inflammatory bowel disease (IBD). We demonstrate in mice that impaired delivery of IL-15 to CD4+ T cells in the colon downmodulates Foxp3 expression (diminishing STAT5 phosphorylation) and enhances RORγt expression (by upregulating the expression of Runx1). In consequence, CD4+ T cells deprived of IL-15 rapidly trigger IBD characterized by enhanced production of pro-inflammatory cytokines (interferon-γ, IL-6) and accumulation of Th1/Th17 cells. Overall, our findings indicate a potentially beneficial role of IL-15 in IBD by fine-tuning the balance between Treg and Th17 cells and controlling intestinal inflammation.

## Abstract (web summary)

Transcription factors directing T cell fate are induced by instructive signals such as cytokines. Here the authors show that IL-15 promotes Foxp3 and inhibits RORγt expression in CD4 T cells, and that IL-15 is critical to suppress colitis by maintaining the Treg to Th1/Th17 ratio in a mouse model.

## Graphical abstract

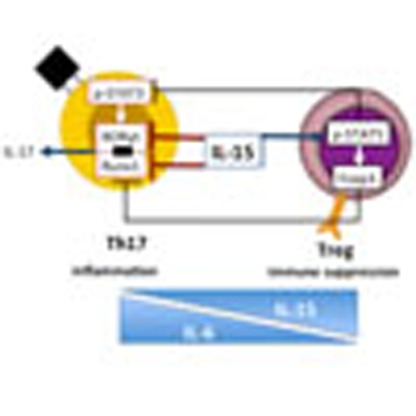

Inflammatory bowel disease (IBD) refers to chronic idiopathic inflammatory disorders affecting the gastrointestinal tract. Two main clinical forms of IBD are Crohn's disease (CD), which can affect any part of the gastrointestinal tract and ulcerative colitis where pathology is restricted to the colonic mucosa^1^.

Conflicting data exist on the potential role of interleukin (IL)-15 in IBD (reviewed in ref. 2). While some reports postulate a pro-inflammatory function of this immune mediator^3^,^4^,^5^other less frequent observations propose a beneficial anti-inflammatory role for IL-15 in IBD^6^,^7^.

Although the importance of IL-15 in proliferation, survival and differentiation of natural killer cells (NK), NK T cells, memory CD8+ T cells, B cells and macrophages is well-documented (reviewed in ref. 8), much less is known about the role of IL-15 in CD4+ T-lymphocyte biology. IL-15 signalling is mediated via the receptor complex composed of the high-affinity IL-15 receptor alpha (IL-15Rα) chain, β chain shared with IL-2 (IL-2/IL-15Rβ) and the common γ (γc) chain^8^,^9^.

IL-15 can stimulate even those target cells that do not express sufficient levels of IL-15Rα in a mechanism of cytokine delivery called trans-presentation. To this end a functional complex of soluble IL-15 and IL-15Rα needs to be formed on the surface of a cytokine-presenting cell, which expresses IL-15Rα and can but does not necessarily have to produce IL-15. This way IL-15 can be provided to a neighbouring cell, which must express both IL-15Rβ and γc receptor chains, for example, like certain T lymphocytes do^10^.

It was demonstrated recently that IL-15 secreted by CD4+ T cells on their activation *in vitro* can downmodulate IL-17 production by these cells in a negative feedback-loop mechanism^11^.

Here we investigated the possible consequences of impaired IL-15 signalling in CD4+ T cells, an important cell subset contributing to IBD^12^.

Peripheral naive CD4+ T lymphocytes are capable to differentiate into various populations of effector cells with specialized function depending on antigen, strength of stimulation, cytokines and other additional factors. This T-cell plasticity probably evolved in the immune system to enable a rapid adaptation to environmental or physiological changes, particularly those occurring in mucosal tissues directly exposed to multiple exogenous antigens (reviewed in ref. 13). T-cell plasticity may, however, enhance the risk of autoimmune diseases, for example, when protective Treg switch their phenotype and function towards self-reactive T helper (Th)-17 cells, like it was reported for psoriasis, autoimmune hepatitis or IBD^14^,^15^,^16^.

Treg cells, which comprise 10–15% of all CD4+ T cells express the forkhead box P3 (Foxp3) transcription factor and high levels of IL-2 receptor alpha (CD25). Treg cells control inflammation in a systemic and local way by counteracting the differentiation of naive CD4+ T cells into effector cells secreting pro-inflammatory cytokines like interferon (IFN)-γ or IL-17, both reported to contribute to autoimmune disorders^17^. IL-17 production requires the expression of the orphan nuclear receptor RORγt typically upregulated in a subset of CD4+ T cells (Th17 cells) after activation in a certain cytokine milieu (transforming growth factor (TGF)-β, IL-6 and IL-23)^18^. Other populations of RORγt-positive cells capable of secreting IL-17 and/or IL-22 are γδ T cells^19^ and a subset of innate lymphoid cells, which do not express any typical lineage-related surface markers^20^. In the light of the so far limited knowledge about CD4+ T-cell plasticity, understanding the conditions and mechanisms that control the fate of two antagonistic cell populations Treg and Th17 cells, is of particular interest, also in the context of developing new therapeutic approaches.

## Results

### Regulatory T cells upregulate RORγt in the absence of IL-15

**Figure 1:**
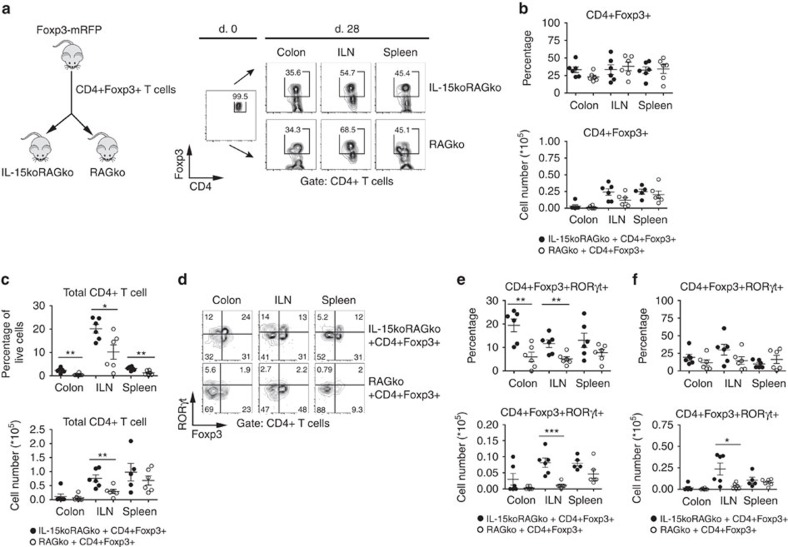
Regulatory T cells acquire RORγt expression on transfer into IL-15-deficient immune-deficient hosts. (**a**) About 2 × 10^5^CD4+Foxp3+ T lymphocytes (Treg) purified from Foxp3-mRFP mice were transferred into RAG2ko or IL-15koRAG2ko mice, which were killed 28 days later: Foxp3 expression by CD4+ T cells before (day 0, left panel) and after transfer in the colon, the inguinal lymph nodes (ILN) and the spleen of injected IL-15koRAG2ko and RAG2ko mice (right panel). (**b**) Percentage among live CD4+ T cells (upper panel) and total number (lower panel) of CD4+Foxp3+ T cells in the colon, the ILN and the spleen of IL-15koRAG2ko and RAG2ko recipients after Treg transfer. (**c**) Frequency among all live cells (upper panel) and number (lower panel) of total CD4+ T cells in the colon, the ILN and the spleen of IL-15koRAG2ko and RAG2ko hosts. (**d**) Expression of Foxp3 and RORγt by CD4+ T cells recovered from the colon, the ILN and the spleen of IL-15koRAG2ko and RAG2ko hosts. (**e**) Percentage among live CD4+ T cells (upper panel) and number (lower panel) of Foxp3+RORγt+CD4+ T cells isolated from the colon, the ILN and the spleen of IL-15koRAG2ko and RAG2ko hosts. (**f**) Percentage among live CD4+ T cells (upper panel) and number (lower panel) of Foxp3-RORγt+CD4+ T cells in IL-15koRAG2ko and RAG2ko hosts. (**b**–**f**) Data compiled from two separate experiments with at least three mice per group. Black circles represent IL-15koRAG2ko hosts, open circles RAG2ko hosts. Each symbol represents an individual mouse. All graphs indicate mean values; error bars denote s.e.m.; *P<0.05; **0.01<*P*<0.05; ***P<0.001(Student's *t*-test values).

While the role of IL-2 on Treg-cell survival has been well-recognized^21^,^22^, less is known about the possible contribution of IL-15, which shares with IL-2 the IL-2Rβ chain critical for IL-2R signalling. We transferred purified populations of Treg cells from Foxp3-mRFP reporter mice^23^ into IL-15-competent or IL-15-deficient, immune-deficient hosts (RAG2ko or IL-15koRAG2ko). Though 99.5% of the initially transferred CD4+ T cells expressed Foxp3, 4 weeks later the frequency of CD4+Foxp3+ T cells recovered from the spleen, colon and inguinal lymph node (ILN) of recipient mice was significantly decreased (Fig. 1a). Nevertheless, the frequency (percentage among all CD4+ T cells) and the absolute number of Foxp3+ cells recovered were comparable in IL-15koRAG2ko and RAG2ko hosts (Fig. 1b), suggesting that IL-15 does not play a critical role in Treg-cell survival. Interestingly, the frequency and absolute number of all CD4+ T lymphocytes and of CD4+Foxp3− population were significantly higher in ILN of IL-15-deficient mice (Fig. 1c and Supplementary Fig. 1c), suggesting that IL-15 may be dispensable for maintaining stable Foxp3 expression, however, it might mediate homeostatic expansion of Foxp3− cells. Phenotypically, in IL-15koRAG2ko hosts a proportion of Foxp3+ T cells concomitantly upregulated RORγt expression (Fig. 1d). These double positive (DP) Foxp3+RORγt+ T cells preferentially accumulated in the colon and ILN of IL-15-deficient mice (Fig. 1e). Moreover, in the IL-15koRAG2ko hosts we detected significantly elevated numbers of Foxp3-RORγt+ T cells in the ILNs (Fig. 1f).

### IL-15 impairs upregulation of RORγt and IL-17 expression

**Figure 2:**
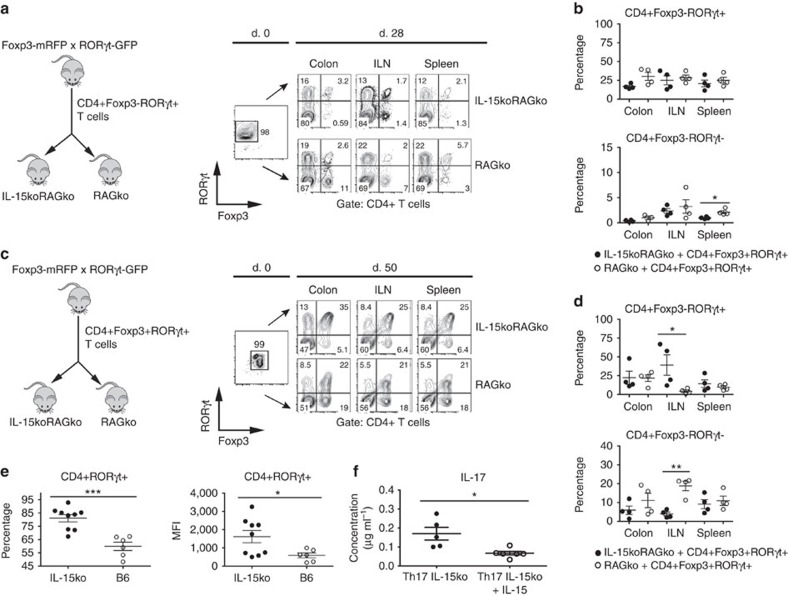
IL-15 modulates the RORγt and Foxp3 expression by CD4+ T cells. (**a**) About 10^5^ RORγt+(Foxp3-) CD4+T lymphocytes from Foxp3-mRFPxRORγt–GFP mice were transferred into RAG2ko or IL-15koRAG2ko mice killed 28 days later: RORγt and Foxp3 expression by CD4+ T cells before (day 0, left panel) and after transfer in the injected RAG2ko and IL-15koRAG2ko mice (day 28, right panel). (**b**) Percentage among live CD4+ T cells of Foxp3-RORγt+CD4+ T cells (upper panel) and Foxp3+RORγt-CD4+ T cells (lower panel) in RAG2ko or IL-15koRAG2ko recipients. (**c**) About 10^5^ Foxp3+RORγt+CD4+T cells from Foxp3-mRFP × RORγt–GFP mice were transferred into RAG2ko or IL-15koRAG2ko mice killed 50 days later: RORγt and Foxp3 expression by CD4+ T cells before (day 0, left panel) and after transfer in RAG2ko or IL-15koRAG2ko hosts (right panel). (**d**) Frequency among live CD4+ T cells of Foxp3-RORγt+CD4+ T cells (upper panel) and Foxp3+RORγt-CD4+ T cells (lower panel) after transfer. (**b**–**d**) Data compiled from two separate experiments with at least two mice per group. Black circles represent IL-15koRAG2ko hosts, open circles RAG2ko hosts. (**e**) Naive CD4+ T lymphocytes from IL-15ko (black circles) or B6 mice (open circles) were cultured 4 days *in vitro* under Th17-polarizing conditions: percentage among live CD4+ T cells (left) and mean fluorescence intensity (MFI, right) of RORγt-expressing CD4+ T cells in the culture. (**f**) Naive CD4+ T lymphocytes from IL-15ko mice were cultured like in **e** in presence or absence of recombinant IL-15: concentration of IL-17 measured by ELISA in cell culture supernatants. (**e**,**f**) Data compiled from two separate experiments with at least three mice per group. Each symbol represents an individual mouse. All graphs indicate means; error bars denote s.e.m.; Student's *t*-test values: *P<0.05; **0.01<*P*<0.05; ***P<0.001).

Since in IL-15koRAG2ko mice a proportion of the injected CD4+Foxp3+ T cells underwent a phenotypic switch towards RORγt-expressing cells, we asked whether IL-15 deficiency might favour expansion of RORγt+ T cells. We injected purified CD4+Foxp3-RORγt+ T cells from double transgenic Foxp3-mRFPxRORγt–green fluorescent protein (Foxp3-mRFPxRORγt–GFP) reporter mice^24^ into IL-15koRAG2ko and RAG2ko mice. We observed that 4 weeks after the transfer, the majority (>70%) of injected CD4+ T cells lost RORγt expression independently of IL-15 availability (Fig. 2a). In the spleen of the IL-15-competent hosts, we detected a significantly higher proportion of CD4+Foxp3+RORγt− cells that lost RORγt and acquired Foxp3 expression (Fig. 2b). To further assess the role of IL-15 in the balance between Foxp3 and RORγt expression by CD4+ T cells, we transferred purified DP CD4+Foxp3+RORγt+ T cells into IL-15-competent and IL-15-deficient hosts. On transfer, only about 30% of the injected CD4+ T cells retained the initial DP expression, whereas the majority lost both Foxp3 and RORγt markers in both hosts (Fig. 2c). Interestingly, the Foxp3-RORγt+ subset was predominant in the ILN of IL-15-deficient hosts, whereas IL-15-competent mice showed preferential accumulation of Foxp3+RORγt− population (Fig. 2d). When unsorted double reporter CD4+ T cells (instead of purified single positive (SP) or DP cells) were transferred into IL-15koRAG2ko and RAG2ko mice, we observed a significant increase in the frequency of RORγt+Foxp3+ DP cells recovered in the IL-15-deficient group (Supplementary Fig. 1a,b). Altogether, the absence of IL-15 may lead to phenotypic changes among CD4+ T cells translated in a reduction of Foxp3 and an increase in RORγt expression.

Our observations suggesting that IL-15 may negatively regulate RORγt expression were confirmed *in vitro*. Naive CD4+T cells purified from wild-type (WT; B6) or IL-15-deficient (IL-15ko) hosts were activated in presence of a Th17-differentiating cytokine mix, which promotes the upregulation of RORγt. We found that the fraction of the CD4+ T cells, which upregulated RORγt in Th17-differentiating conditions, was significantly elevated in IL-15-deficient cells and these cells expressed significantly higher levels of RORγt than conventional CD4+ T cells (Fig. 2e). IL-17 production by IL-15-deficient Th17 cells seemed to be slightly elevated as compared with WT B6 Th17 cells, however, the difference did not reach the level of statistical significance, while IL-15 was not detectable in this setting (Supplementary Fig. 2c).

Nevertheless, we observed that the production of IL-17 by IL-15ko-Th17-differentiated cells was significantly lower when these cells were grown in presence of recombinant IL-15 (Fig. 2f). Addition of rmIL-15 to WT B6 Th17 cells had no significant impact on IL-17 production probably due to autocrine secretion of IL-15 by these cells as previously reported^11^ (Supplementary Fig. 2c,d). These findings indicate that both *in vivo* and *in vitro* IL-15 could negatively regulate RORγt expression by CD4+ T cells. In contrast to Th17 differentiation programme, Th1 and Treg differentiation *in vitro* was not affected by the absence of IL-15 (Supplementary Fig. 2a,b).

### Impaired delivery of IL-15 to CD4+ T cells results in IBD

**Figure 3:**
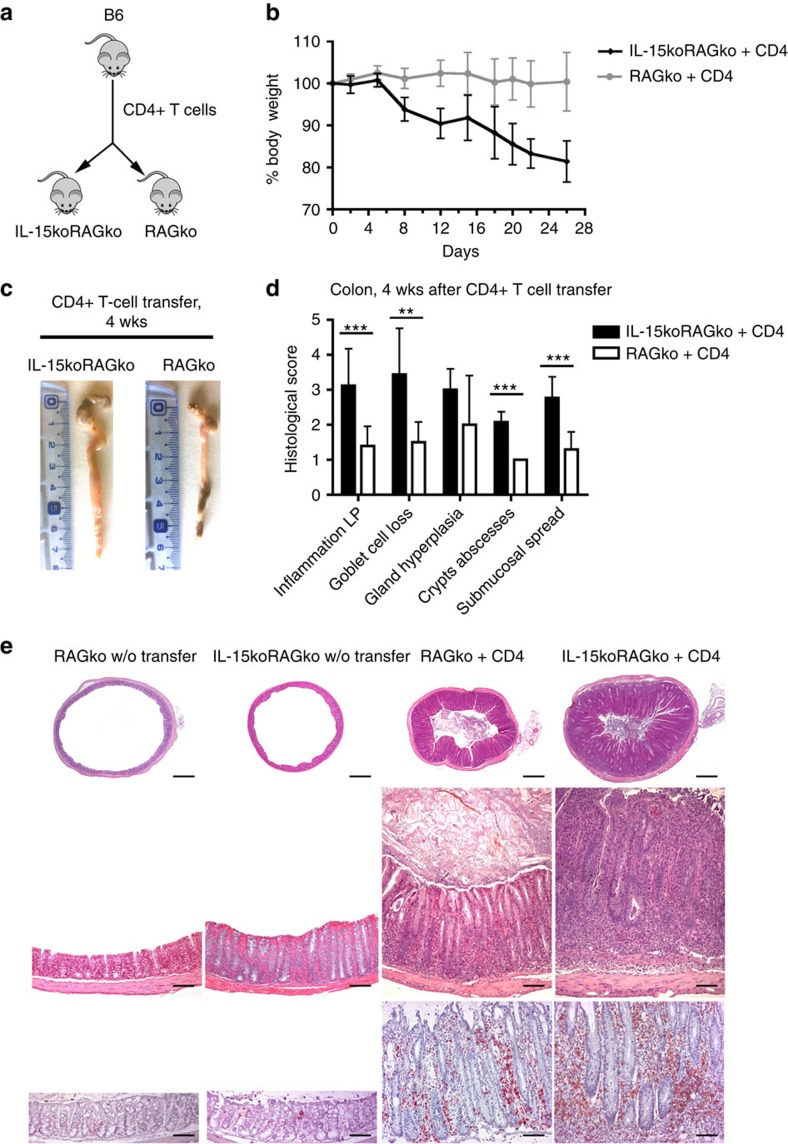
Impaired IL-15 signalling results in accelerated colitis in immune-deficient hosts following CD4**+** T-cell transfer. (**a**) About 2 × 10^5^ CD4+ T lymphocytes from B6 mice were transferred into RAG2ko or IL-15koRAG2ko mice killed 4 weeks later. (**b**) Body weight curve of recipient RAG2ko (grey line) and IL-15koRAG2ko mice (black line). (**c**) Representative images of colons from RAG2ko and IL-15koRAG2ko recipients (*n*>15 per group). (**d**) Histological scores (from 0—minimal to 5—severe) of microscopic changes in the colon of RAG2ko (white bars) and IL-15koRAG2ko mice (black bars) after transfer. Data compiled from three separate experiments with at least three mice per group. All graphs indicate means; error bars denote s.e.m.; Student's *t*-test values: **0.01<*P*<0.05, ***P<0.001. (**e**) Upper and middle panels: haematoxylin and eosin-stained sections of colons from RAG2ko and IL-15koRAG2ko mice (*n*=6 per group) before (left panel) and after CD4+ T-cell transfer (right panel) Bottom panel: immunostaining for CD4+ T cells. Original magnification: upper panel × 4 (scale bar, 250 μm), middle and bottom panels × 10 (scale bar, 100 μm).

To investigate the consequences of the IL-15-mediated CD4+ T-cell plasticity in IBD, we transferred total CD4+ T cells isolated from WT B6 mice into either RAG2ko or IL-15koRAG2ko hosts (Fig. 3a). In IL-15-deficient host mice, we observed a premature intestinal inflammation resembling IBD in humans, that is, body weight loss and marked enlargement of the large intestine at necropsy (Fig. 3b,c). Histological analysis of colonic samples from IL-15-deficient recipients showed severe and extensive infiltration of inflammatory cells including CD4+ T cells in the lamina propria (LP), with a spread of inflammation to the deeper layers of the colonic wall. Other microscopic findings (that is, goblet cell loss, crypts abscesses, submucosal spread) were also severe in IL-15-deficient mice (Fig. 3d,e) and not observed in IL-15-sufficient RAG2ko group.

Similarly, adoptive transfers of CD4+ T cells from donors lacking IL-15Rα (IL-15Rαko) into IL-15-suficient RAG2ko hosts resulted, 4 weeks later, in an IBD-like pathology (Supplementary Fig. 3a,b). When the both adoptively transferred CD4+ T cells and the hosts lacked IL-15Rα, the destruction of the colon mucosa was even more severe and more extensive (that is, reaching the submucosa)(Supplementary Fig. 3c,d). These observations led us to the conclusion that delivery of IL-15 to differentiating CD4+ T cells may have a protective role for colon mucosa.

We speculated whether IL-6 neutralization^25^ could protect IL-15-deficient mice from premature colitis. We monitored development of colitis after transfer of total CD4+ T cells in IL-15koIL-6koRAG2ko mice compared with RAG2ko, IL-6koRAG2ko and IL-15koRAG2ko recipients (Supplementary Fig. 4a). While IL-15koRAG2ko mice had to be killed due to the rapid body weight loss within 4 weeks post transfer, the remaining groups of hosts did not show remarkable pathological changes up to 9 weeks post transfer (Supplementary Fig. 4b,c), demonstrating that the severe colitis induced by T cells in absence of IL-15 was not observed in the absence of IL-6.

### Treg with impaired IL-15 signalling have diminished function

**Figure 4:**
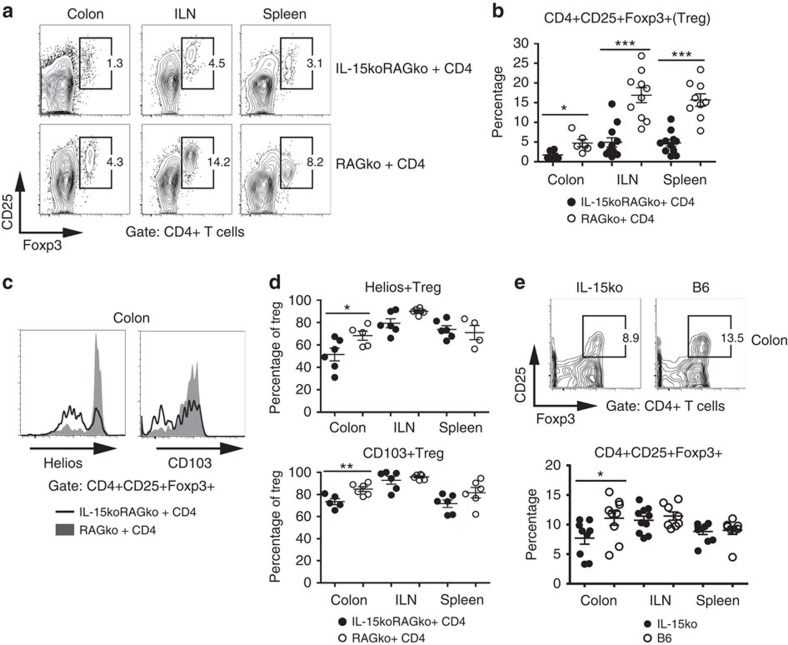
IL-15koRAG2ko mice with colitis have diminished percentage and altered phenotype of Treg. (**a**–**e**) About 2 × 10^5^ CD4+ T lymphocytes from B6 mice were transferred into RAG2ko or IL-15koRAG2ko mice killed 4 weeks later. (**a**) Expression of CD25 and Foxp3 on CD4+ T cells in the injected IL-15koRAG2ko and RAG2ko mice. (**b**) Frequency among live CD4+ T cells of CD4+CD25+Foxp3+ T cells (Treg) recovered from adoptively transferred IL-15koRAG2ko and RAG2ko mice. (**c**) Expression of Treg-related markers: Helios and CD103 by Treg cells isolated from the colon of IL-15koRAG2ko (black line) and from RAG2ko recipient mice (grey shadow). (**d**) Frequency among Treg (CD4+CD25+Foxp3+) of Helios- (upper panel) and CD103 (lower panel) -expressing cells recovered from the injected IL-15koRAG2ko and RAG2ko mice. (**b**–**d**) Open circles RAG2ko mice; black circles: IL-15koRAG2ko mice. (**e**) Expression of CD25 and Foxp3 on CD4+ T cells in the colon of unmanipulated IL-15ko or B6 mice (upper panel) and frequency among live CD4+ T cells of CD4+CD25+Foxp3+ Treg in the colon, the ILN and the spleen of unmanipulated IL-15ko or B6 mice (lower panel). Data compiled from two separate experiments with at least three mice per group. Each symbol represents an individual mouse. All graphs indicate means; error bars denote s.e.m.; Student's *t*-test values: *P<0.05; ***P<0.001.

Taking into account that IL-15-deficient mice developed severe colon inflammation despite the presence of Treg cells among the adoptively transferred CD4+ T cells, we investigated the fate of the transferred Treg cells in IL-15-deficient mice with colitis. We noticed a lower frequency of CD4+CD25+Foxp3+ Treg cells in the colon, ILN and spleen of IL-15-deficient mice as compared with RAG2ko group (Fig. 4a,b). In addition, the Treg population isolated from the inflamed colon tissue of IL-15koRAG2ko hosts had significantly decreased expression of Treg activation and function markers, that is, Helios and CD103 (Fig. 4c,d). Of note, the observation, that IL-15 in addition to IL-2 may modulate the percentage of Treg *in vivo*, was further confirmed in unmanipulated IL-15ko and IL-15Rαko mice which demonstrated significantly lower frequencies of Treg cells locally in the colon as compared with WT B6 mice (Fig. 4e and Supplementary Fig. 5). Since Treg population is dependent on IL-2 secreted mainly by other T cells^26^, we speculate that in peripheral tissues where T cells are less frequent than in lymphoid organs, IL-15, produced by a variety of non-immune cells including epithelial cells^8^, could play a role in Treg function. Thus, in the absence of IL-15, Treg cells undergo phenotypic and functional changes in the colon and in consequence fail to curtail an inflammatory response.

### Regulatory T cells deprived of IL-15 fail to prevent IBD

**Figure 5:**
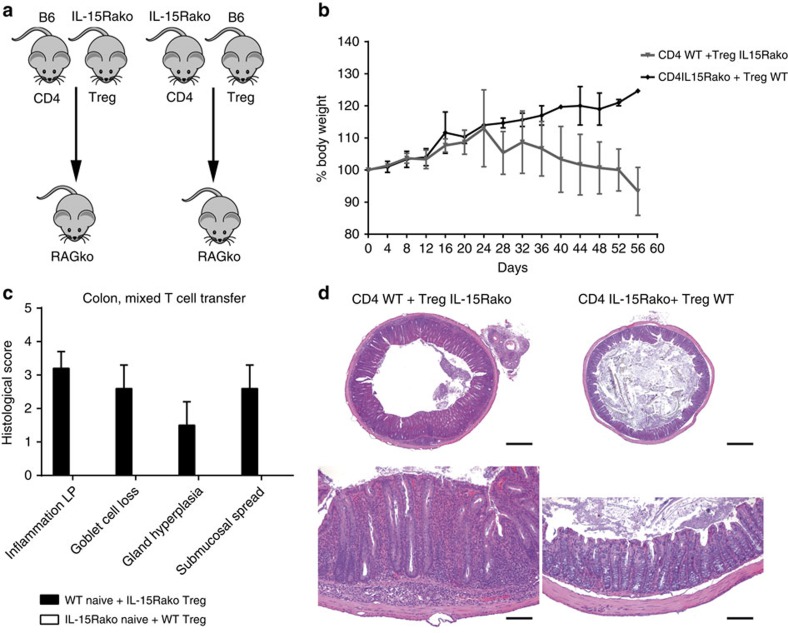
Treg with impaired IL-15 signaling fail to prevent colitis in RAG2ko hosts injected with both Treg and naive CD4+ T cells. (**a**) Treg (CD4+Foxp3+RORγt−) and naive CD4+ T lymphocytes (CD4+Foxp3-CD62L^hi^CD45RB^hi^ ) were sorted from Foxp3-mRFP × RORγt–GFP and from IL-15Rαko × Foxp3-mRFP × RORγt–GFP mice. About 10^5^ IL-15Rαko Treg cells were injected together with 10^5^ IL-15-competent naive CD4+ T cells into RAG2ko mice. Similarly, 10^5^ IL-15Rαko naive CD4+ were injected together with 10^5^ IL-15-competent Treg cells into other group of RAG2ko mice. Recipient mice were killed 8 weeks later. (**b**) Body weight curve of recipient RAG2ko, which received IL-15Rαko Treg and WT CD4+ T cells (grey line) and of RAG2ko mice injected with IL-15Rαko CD4+ and WT Treg cells (black line). (**c**) Histological scores (from 0—minimal to 5—severe) of microscopic changes in the colon of RAG2ko mice following transfer of IL-15Rαko Treg and WT CD4+ T cells (black bars) and RAG2ko following transfer of IL-15Rαko CD4+ and WT Treg cells (white bars). The score values for the second group are 0, therefore they are not visible 8 weeks post transfer. All graphs indicate mean values; error bars denote s.e.m. (**d**) Haematoxylin and eosin-stained sections of colons from RAG2ko mice after naive CD4+/Treg T-cell transfer. Original magnification: upper panel × 4 (scale bar, 250 μm), bottom panel × 10 (scale bar, 100 μm). (**a**–**d**) Data compiled from two separate experiments with at least three mice per group.

To evaluate the suppressor potential of Treg cells with defective IL-15 signalling *in vivo*, we performed adoptive transfers of mixed Treg: naive CD4+ T-cell populations (1:1) into RAG2ko hosts. In the first group we combined IL-15Rαko Treg with IL-15-competent (WT) naive CD4+ T cells. In the control group, WT Treg cells were co-transferred with IL-15Rαko naive CD4+ T lymphocytes (Fig. 5a). While WT Treg cells efficiently prevented possible intestinal inflammation mediated by IL-15Rαko naive CD4+ T cells, the mice injected with IL-15-deprived Treg and WT CD4+ T cells demonstrated significant body weight loss and symptoms of colitis (Fig. 5b). Histological analysis of colonic samples from recipients transferred with IL-15Rαko Treg showed severe and extensive infiltration of inflammatory cells in the LP, severe goblet cell loss, crypts abscesses and submucosal spread while these findings were absent in the group injected with WT Treg (Fig. 5c,d).

### Th1/Th17 cells expand on colon inflammation in IL-15ko hosts

**Figure 6:**
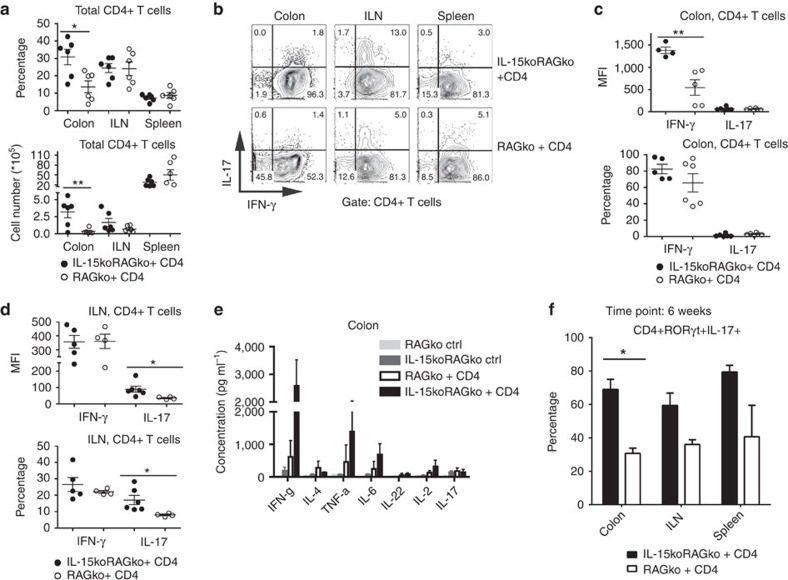
Adoptively transferred CD4+ T cells secrete IFN-γ and IL-17 and contribute to colon inflammation. About 2 × 10^5^ CD4+ T lymphocytes purified from B6 mice were transferred into RAG2ko or IL-15koRAG2ko mice, which were killed 4 (**a**–**e**) or 6 (**f**) weeks later. (**a**) Frequencies among all live cells (upper panel) and absolute numbers (lower panel) of CD4+ T cells in the colon, the ILN and the spleen of IL-15koRAG2ko and RAG2ko hosts. (**b**) Expression of IFN-γ and IL-17 by CD4+ T cells isolated from adoptively transferred IL-15koRAG2ko and RAG2ko mice. (**c**) Median fluorescence intensity (MFI, upper panel) and frequencies among live CD4+ T cells (lower panel) of IFN-γ- and IL-17-expressing cells in the colon of IL-15koRAG2ko and RAG2ko recipients. (**d**) MFI values (upper panel) and frequencies among live CD4+ T cells (lower panel) of IFN-γ- and IL-17-expressing cells in the ILN of IL-15koRAG2ko and RAG2ko mice. (**e**) Concentration of cytokines (pg ml^−1^) secreted in the culture of lamina propria cells isolated from the colon of untreated RAG2ko mice (light grey bars), untreated IL-15koRAG2ko (dark grey bars), RAG2ko mice after CD4+ T-cell transfer (white bars) and IL-15koRAG2ko mice after CD4+ T-cell transfer (black bars). (**f**) Frequencies among live CD4+ T cells of RORgt+IL-17+ cells in the colon of IL-15koRAG2ko and RAG2ko mice 6 weeks post transfer. (**a**–**f**) Data compiled from two separate experiments with a minimum of three mice per group; each dot represents an individual mouse. All graphs indicate means; error bars denote s.e.m.; Student's *t*-test values: *P<0.05; **0.01<*P*<0.05.

Accompanying the modifications in Treg status in IL-15koRAG2ko mice, which develop premature colitis following CD4+ T-cell transfer, we have found that the frequency and absolute number of total CD4+ T cells were significantly higher in the colon of IL-15-deficient mice (Fig. 6a). Moreover, in line with reports indicating that IFN-γ and IL-17 are the main immune mediators in Th1/Th17-type IBD^12^ and that Th17 cells are plastic and can secrete not only IL-17-type cytokines but also IFN-γ (ref. 27), we found significantly higher mean fluorescence intensity values for IFN-γ and IL-17 production in the colon and ILN of IL-15-deficient mice with colitis (Fig. 6b–d). In addition, the concentration of pro-inflammatory cytokines, in particular IFN-γ, TNF-α and IL-6 secreted by total LP cells in the colon, was two- to sixfold higher in IL-15-deficient mice with CD4+ T-cell-driven colitis compared with RAG2ko host mice (Fig. 6e). In colons of IL-15koRAG2ko mice which developed intestinal inflammation, we also detected significantly higher frequencies of RORγt+IL-17+ CD4+ T cells as compared with healthy RAG2ko mice (Fig. 6f). This finding further underlines the importance of IL-17- and RORγt-mediated inflammation in IL-15-deficient mice.

### IL-15ko CD4+ T cells downregulate p-STAT5 and upregulate Runx1

**Figure 7:**
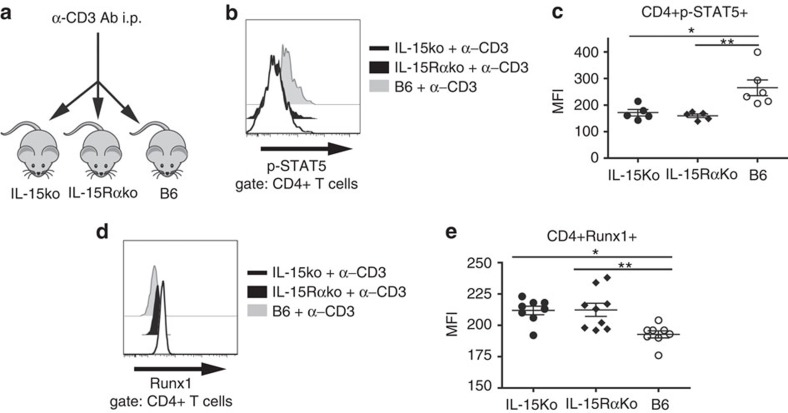
CD4+ T cells with impaired IL-15 signaling respond to TCR stimulation by down-regulation of pSTAT5 and up-regulation of Runx1. (**a**) IL-15ko, IL-15Rαko (IL-15 receptor alpha deficient) and B6 mice received i.p. 50 μg α-CD3 Ab and were killed 2 h later. (**b**) Expression of p-STAT5 (phosphorylated signal transducer and activator of transcription 5) on CD4+ T cells in the LN sampled from the injected mice and re-stimulated 20 min *in vitro* with recombinant mouse (rm) IL-15. (**c**) Median fluorescence intensity (MFI) of p-STAT5 expression on CD4+ T cells isolated from the injected mice and re-stimulated 20 min *in vitro* with rmIL-15. (**d**) Expression of Runx1 (runt-related transcription factor-1) in CD4+ T cells isolated from the LN of IL-15ko, IL-15Rαko and B6 mice 2 h after injection of α-CD3 Ab. (**e**) MFI of Runx1 expression on CD4+ T cells isolated from the injected mice. Black line: IL-15ko, black shadow: IL-15Rαko, grey shadow: B6. Black circles: IL-15ko, black diamonds: IL-15Rαko, open circles B6. (**b**–**e**) Data compiled from two separate experiments with at least two mice per group. Each symbol represents an individual mouse. One-way analysis of variance (*P*<0.01), Tukey's post-test values: *P<0.05; **0.01<*P*<0.05.

To compare the balance between Treg versus Th17 differentiation programmes shortly after activation of CD4+ T cells *in vivo*, we triggered an overall CD4+ T-cell activation in IL-15-competent and -deficient mice by injecting B6, IL-15ko, IL-15Rαko and mice with α-CD3 Ab (Fig. 7a). Two hours later, we evaluated the expression of phosphorylated (p-) STAT5 and runt-related transcription factor-1 (Runx1), that is, transcription factors related to Treg and Th17 phenotype, respectively^28^,^29^, by CD4+ T cells isolated from the LN of the injected mice. Transcription of Foxp3 and the resulting Treg induction are promoted by the signalling transmitted through p-STAT5 which acts downstream of the common IL-2/IL-15Rβ chain^30^,^31^. Here CD4+ T cells with disrupted IL-15 signalling showed significantly lower expression of p-STAT5 as compared with B6 cells (Fig. 7b,c). This observation may explain the diminished percentages of Foxp3+ Treg cells observed in the colon of IL-15ko and IL-15Rαko mice, as well as in IL-15koRAG2ko mice with colitis (Fig. 4b,e and Supplementary Fig. 5). In contrast, the expression of Runx1, a transcription factor known to act cooperatively with RORγt required for Th17 differentiation^29^, was significantly elevated in CD4+ T cells with impaired IL-15 signalling as compared with B6 cells (Fig. 7d,e). This finding is in agreement with the preferential acquisition of RORγt expression on CD4+ T cells deprived of IL-15, which we observed both *in vivo* following adoptive transfers, and *in vitro* in the culture of IL-15-deficient CD4+ T cells under Th17-differentiating conditions (Fig. 2). In steady state (without *in vivo* α-CD3 stimulation or without IL-15 re-stimulation *in vitro*), we did not observe any significant difference in p-STAT5 or Runx1 expression by CD4+ T cells in IL-15-deficient versus WT mice. The same was true for *in vitro*-generated Th1, Th17 or Treg cells (Supplementary Fig. 6).

## Discussion

Immune responses in the intestine need to be tightly controlled to maintain a balance between effector and regulatory pathways. Changes in this balance, for example, due to the impaired Treg function, may result in several forms of chronic intestinal inflammation like IBD. We now report that CD4+ T-cell transfer into immune-deficient mice with disrupted IL-15 signalling led to the development of a severe and accelerated form of IBD, resembling CD, both in the histological features as well as in the Th1/Th17 cytokine profile^12^. Taking into account that Th17 and Treg cells were reported to play a key role in IBD^1^,^32^, we investigated the stability of RORγt and Foxp3 expression by CD4+ T cells in IL-15-deficient hosts. We found that following transfer into IL-15-deficient immune-deficient hosts, Foxp3+RORγt− Treg cells upregulated RORγt independently of whether or not they maintained Foxp3 expression. In line with this finding we also show that DP CD4+Foxp3+RORγt+ T cells, the subset previously described in patients suffering from colon cancers^33^, preferentially differentiated into SP CD4+Foxp3+RORγt− T cells in presence of IL-15, and into SP CD4+Foxp3-RORγt+ T cells in IL-15-deficient immune-deficient mice. Since we did not observe any increased accrual of committed CD4+RORγt+ T cells in IL-15-deficient mice, we concluded that IL-15 does not have a direct role on the survival of CD4+RORγt+ T cells but may modulate the balance between Foxp3 and RORγt expression in uncommitted CD4+ T cells and shift it towards Foxp3 expression.

Recently Pandiyan *et al*.^11^ concluded from *in vitro* data that IL-15 could have a negative regulatory role in fine-tuning IL-17 production. We observed that *in vitro*, the addition of recombinant IL-15 to the culture of Th17 (CD4+RORγt+) T cells from IL-15ko mice diminished the secretion of IL-17A. While others proposed that the IL-15-mediated inhibition of IL-17 is p-STAT5- and not RORγt-dependent^11^, we found that IL-15-deficient cells significantly did upregulate RORγt expression as compared with B6 cells. This finding is in line with our *in vivo* data on IL-15-dependent regulation of RORγt expression.

RORγt and Runx1 transcription factors were reported to act cooperatively to promote Th17 differentiation^29^. However, the expression of RORγt and Runx1 can be efficiently repressed by Foxp3 which acts to prevent the differentiation of Th17 cells^34^. Here we now report the link between IL-15 and Runx1 expression by demonstrating *in vivo* that IL-15 deficiency leads to significantly elevated expression of Runx1 on activated but not on naive CD4+ T cells. Since Runx1 is a key molecule that simultaneously orchestrates the expression of RORγt and Foxp3 in CD4+ T cells^34^, our findings could explain the mechanism of the imbalance between Th17 and Treg cells reported in IL-15koRAG2ko hosts.

As a consequence of the imbalanced Th17/Treg-cell plasticity, mice with disrupted IL-15 signalling developed on transfer of CD4+ T cells from WT donors a rapid form of colon inflammation resembling CD. In IL-15-deficient hosts, proliferating effector Th1/Th17 cells rapidly outnumbered Treg cells, which underwent phenotypic changes in the colon and failed to curtail the intestinal inflammation. Of note, we found a local decrease of Treg frequencies not only in IL-15koRAG2ko mice with colitis but also in the colon of unmanipulated IL-15ko and in IL-15Rαko mice. In addition, we demonstrated *in vivo* that Treg cells with impaired IL-15 signalling when co-transferred with WT naive CD4+T cells into RAG2ko mice failed to prevent the development of colitis, while WT Treg co-transferred with IL-15Rαko naive CD4+ T cells efficiently protected against IBD. These observations indicate that IL-15 may play a complementary role to IL-2 in promoting Treg-cell stability. Previous reports indicated that *in vitro* IL-15 induced CD25 and Foxp3 expression in human cells^35^ and that IL-15 presentation in trans induced Treg proliferation^36^. Moreover, cytokines signalling via STAT5, including IL-15, upregulate Foxp3 expression^28^. We show that *in vivo*, CD4+ T cells in the presence of disrupted IL-15 signalling (IL-15ko and IL-15Rαko) showed significantly lower expression of p-STAT5 following TCR stimulation (but not in the steady state). In the absence of IL-15, the impaired p-STAT5 signalling could diminish transcription of Foxp3, which stabilizes Treg lineage and suppresses Th17 differentiation^30^, thus resulting in the lower Treg frequencies observed in the colon of unmanipulated IL-15ko and IL-15Rαko mice and also in IL-15koRAG2ko mice with Th1/Th17-type colitis. In contrast to IL-2, which is produced only by haematopoietic cells, mainly CD4+ T cells^37^, IL-15 is secreted by many different cell types^8^ and therefore can play a decisive role in Treg-cell maintenance and function in sites were IL-2 accessibility is poor due to low frequencies of T cells, for example, colon and other peripheral tissues.

To clarify the role of the pro-inflammatory milieu in the induced colitis, we investigated the potential interplay between IL-15 and IL-6, the latter known as the key cytokine involved in Th17 differentiation via induction of STAT3 and RORγt (ref. 38). We found that the mice deficient for both IL-6 and IL-15 (IL-15koIL-6koRAG2ko) were resistant to T-cell-induced colitis in contrast to IL-15koRAG2ko mice. Thus, IL-6 and IL-15 seem to have the opposite effects on CD4+ T-cell differentiation in development of IBD in a widely accepted model^32^,^39^.

In contrast to our observations, previous studies based on the enhanced expression of IL-15 in IBD patients have proposed a pro-inflammatory role for IL-15 (refs 3, 40). Nevertheless, there are contradictory observations that postulated anti-inflammatory, IL-15-mediated inhibition of IFN-γ and TNF-α secretion in patients with CD^7^, and reported that endogenous IL-15 could protect the intestinal epithelium and thus prevent from extensive mucosal damage in IBD^6^. These observations are in line with our data, where IL-15-deficient mice or mice with abrogated IL-15 signalling developed after CD4+ T-cell transfer intestinal inflammation with prominent damage of the mucosa. Thus, our current observations support a disregarded potentially beneficial role for IL-15 in IBD.

In conclusion, our findings demonstrate *in vivo* that IL-15 mediated positively the regulation of Foxp3 by enhanced p-STAT5 signalling and supported Treg-cell stability, while reciprocally it induced the negative regulation of RORγt and Runx1 expression associated with Th17 cells (Supplementary Fig. 7). More importantly, we found that IL-15-mediated imbalance of T-cell plasticity, that is, loss of Treg control over expanding Th1/Th17 cells, resulted in severe intestinal inflammation resembling IBD in humans. Our findings could help to solve the current controversies and impact future directions of research and therapeutic approaches in IBD patients.

## Methods

### Mice

C57Bl/6, IL-15koRAG2ko, IL-6koRAG2ko, IL-15koIL-6koRAG2ko, IL-15Rαko, IL-15ko, IL-15RαkoRAG2ko, Foxp3-mRFP, Foxp3-mRFPxRORγt–GFP and RAG2ko mice (all on B6 background) were bred and kept in the same Animal Facility of the Institut Pasteur. Male and female mice aged 8–12 weeks were used for experiments. All the animal experiments and handling procedures were approved and performed according to the guidelines of the Pasteur Institute Safety Committee in accordance with French and European guidelines and the Ethics Committee of Paris.

### Adoptive transfers

Spleen and LN cells from B6 mice or where indicated from IL-15 Rαko mice were enriched for CD4+ T lymphocytes by negative selection using the CD4 T-cell isolation kit and autoMACS-Pro separator (Miltenyi Biotec, Germany). For other transfers, spleen and LN cells from Foxp3-mRFP or Foxp3-mRFPxRORγt–GFP mice were first enriched for CD4+ T cells as above and CD4+mRFP+(Foxp3+), CD4+GFP+(RORγt+) or CD4+mRFP+(Foxp3+) GFP+(RORγt+) cells purified using a BD FACS AriaII cell sorter (Becton Dickinson, San Jose, USA). For adoptive transfer 10^5^–4 × 10^5^ purified CD4+ T cells were injected i.v. usually into IL-15koRAG2ko or RAG2ko (unless otherwise stated), which were killed 4–9 weeks later.

### Flow cytometry

All anti-mouse fluorescent monoclonal antibodies (mAbs) used in this study were used at concentrations recommended by the manufacturer and according to manufacturer's instructions. Following mAbs were from BD Biosciences (San Jose, USA): α-CD4 (ref. 557956),α-CD3 (ref. 558214), α-TCR-β (ref. 562839), α-CD103 (ref. 557494), α-CD25 (ref. 552880),CD45RB (ref. 562848), CD62L (ref. 553152) and from e-Bioscience (San Diego, USA): α-IFN-γ (ref. 25–7311), α-IL-17 (ref. 12–7177), α-Helios (ref. 17–9883), α-Foxp3 (ref. 35–5773) and α-RORγt (ref. 12–6988).

For intracellular cytokine staining, 10^6^ cells per ml were incubated for 4 h at 37 °C with phorbolmyristate acetate (PMA) (10 ng ml^−1^), ionomycin (100 ng ml^−1^) and brefeldin A (10 μg ml^−1^) (all Sigma-Aldrich, Germany) in RPMI medium complemented with 10% FCS (Gibco, USA). Surface staining with α-CD4, α-CD3 or α-TCR-β (ref. above) was followed by fixation for 20 min with 2% paraformaldehyde, next by permeabilization with 0.01% Igepal CA-630 (Sigma-Aldrich) and by intracellular staining for 30 min with anti-cytokine antibodies: α-IFN-γ or α-IL-17.

In case of p-STAT5 and Runx1 staining, mice were first injected i.p. with 50 μg α-CD3 Ab and killed 2 h later. Cells isolated from pooled MLN and ILN were first stained with α-CD4 APC Ab followed by intracellular staining either with α-p-STAT5 Alexa Fluor-647 Ab (ref. 612599; BD Biosciences) or with α-Runx1 primary Ab (ref. sc-8564; Santa Cruz Biotechnology, Texas, USA); the latter in combination with α-goat IgG PE secondary Ab (ref. ab7007; Abcam, UK) according to manufacturer's instructions. All cell samples stained with fluorescent Abs were measured using a flow cytometer (FACS LSR Fortessa, BD, USA).

### Isolation of LP cells from the colon

After removing faeces and impurities by flushing with PBS, colon was opened longitudinally and cut into small pieces, which were then incubated twice in HBSS medium+2 mM EDTA to remove epithelium and next in pure HBSS, each time 15 min/37 °C with constant mixing. Then, colon tissue was incubated 15 min/37 °C with constant mixing in RPMI medium+20% FCS+0.6 μg ml^−1^ collagenase (Sigma-Aldrich) to release LP cells from the surrounding tissue and was filtered through a 40-μm nylon mesh. Supernatant containing LP cells was collected and stored on ice, whereas the remaining tissue pieces were again digested and filtered as described in the last step. Supernatants collected in both steps were pooled and spin down. The resulting cell pellet was suspended in 75% Percoll and next overlaid carefully with 40% Percoll. After centrifugation (without brakes, 15 min, 750 g, 20 °C) LP cells were collected from the interphase ring, then washed with RPMI medium and used for Ab staining (modified from ref. 41).

### Determination of cytokine concentration in the colon

LP cells isolated as described above from colons of either non-manipulated or adoptively transferred RAG2ko and IL-15koRAG2ko mice (4 weeks after transfer with B6 CD4+ T cells) were cultured *in vitro* in RMPI+10% FCS and stimulated 4 h with PMA/ionomycin. Supernatants collected from LP cell culture were subsequently tested for presence of 11 cytokines with mouse FlowCytomix kit (e-Bioscience) according to the manufacturer's instructions.

### Differentiation of Th17 cells *in vitro*

Naive CD4+ T cells (CD25^neg^CD62L^hi^CD45RB^hi^) purified from spleen and LN of B6 or IL-15ko mice (FACS AriaII) were cultured in a cell culture plate coated with α-CD3Ab-(ref. 16–0031) at 2 × 10^6^ cells per ml in RPMI+10% FCS supplemented with α-CD28 (ref. 16–0281), TGF-β (ref. 14–8342), IL-6 (ref. 14–8061), IL-23 (ref. 14–8231), α-IFN-γ (ref. 16–7411), α-IL-4 (ref. 16–7041) and where indicated with rmIL-15 (ref. 14–8153; all reagents from e-Bioscience). After 4 days, the cells were stained for RORγt expression. Cell culture supernatants were tested for IL-17 concentration by ELISA kit (PeproTech, Rocky Hill, USA).

### Histological analysis and scoring

After euthanasia, the entire colon was sampled, fixed in 10% neutral buffered formalin and embedded in paraffin. Three transversal 4-mm paraffin sections, taken from the proximal, mid and distal third of the colon, were stained with haematoxylin and eosin and Alcian Blue for mucus. All sections were evaluated microscopically and following histological findings were scored semi-quantitatively: inflammation of the LP, goblet cell loss (on Alcian Blue staining), gland atrophy, crypt abscesses, gland hyperplasia and submucosal spread. A five-grade scale was applied to histological findings (0: no lesions, 1: minimal, 2: mild, 3: moderate; 4: marked; 5: severe). Submucosal spread of the inflammation was given a score ranging 0–3 (0: no extension to the submucosal layer, 1: extension to the submucosal layer, 2: extension to the mesentery). For immunohistochemistry, the colon was fixed in JB fixative (zinc acetate 0.5%, zinc chloride 0.05% and calcium acetate 0.05% in Tris buffer at pH 7) for 48 h and then embedded in low-melting point paraffin (poly ethylene glycol distearate; Sigma, USA). Three transversal sections from the proximal, mid and distal part of the colon were labelled using a rat anti-mouse α-CD4 antibody (BD).

### Generation of Th1 cells *in vitro*

Naive CD4+ T cells (CD25^neg^CD62L^hi^CD45RB^hi^) purified from spleen and LN of B6 or IL-15ko mice (FACS AriaII) were cultured 5 days in a α-CD3Ab-coated plate at 2x10^6^ cells per ml in RPMI+10% FCS supplemented with IL-2 (ref. 34–8021), IL-12 (ref. 34–8121), α-CD28 and α-IL-4 (ref. above; all reagents from e-Bioscience) in presence of irradiated T-cell-depleted spleen cells as described by Fitch *et al*.^42^.

### Generation of Treg *in vitro*

In the first step, total CD4+ T cells were purified from spleen and LN of B6 or IL-15ko mice by negative magnetic sorting with CD4 cell isolation kit according to the manufacturer's instructions. Next, the enriched CD4+ T cells were labelled with α–CD25 PE Ab (ref. 130102788) and then incubated with anti-PE Ab conjugated with microbeads (ref. 130–048–801; all reagents from Miltenyi Biotec). CD4+CD25− T cells were purified by negative selection and were cultured 5 days in a α-CD3Ab-coated plate at 2 × 10^6^ cells per ml in RPMI+10% FCS supplemented with α-CD28 and TGF-β as described by Fantini *et al*.^43^

### Statistical analysis

*P* values were calculated using GraphPad Prism software with unpaired, two-tailed Student's *t*-test or where indicated with one-way analysis of variance followed by Tukey's test.

## Additional information

**How to cite this article:** Tosiek, M. J. *et al*. IL-15-dependent balance between Foxp3 and RORγt expression impacts inflammatory bowel disease. *Nat. Commun.* 7:10888 doi: 10.1038/ncomms10888 (2016).

## Supplementary Material

Supplementary Information Supplementary Figures 1-7
