# Supplementary material for: IL-15-dependent balance between Foxp3 and RORγt expression impacts inflammatory bowel disease
Source: Nat Commun. 2016 Mar 11;7:10888. doi: 10.1038/ncomms10888 (PMC4792960; doi:10.1038/ncomms10888)
Supplement: Supplementary Information — Supplementary Figures 1-7 [file ncomms10888-s1.pdf]

Supplementary Figures

Supplementary Figure 1

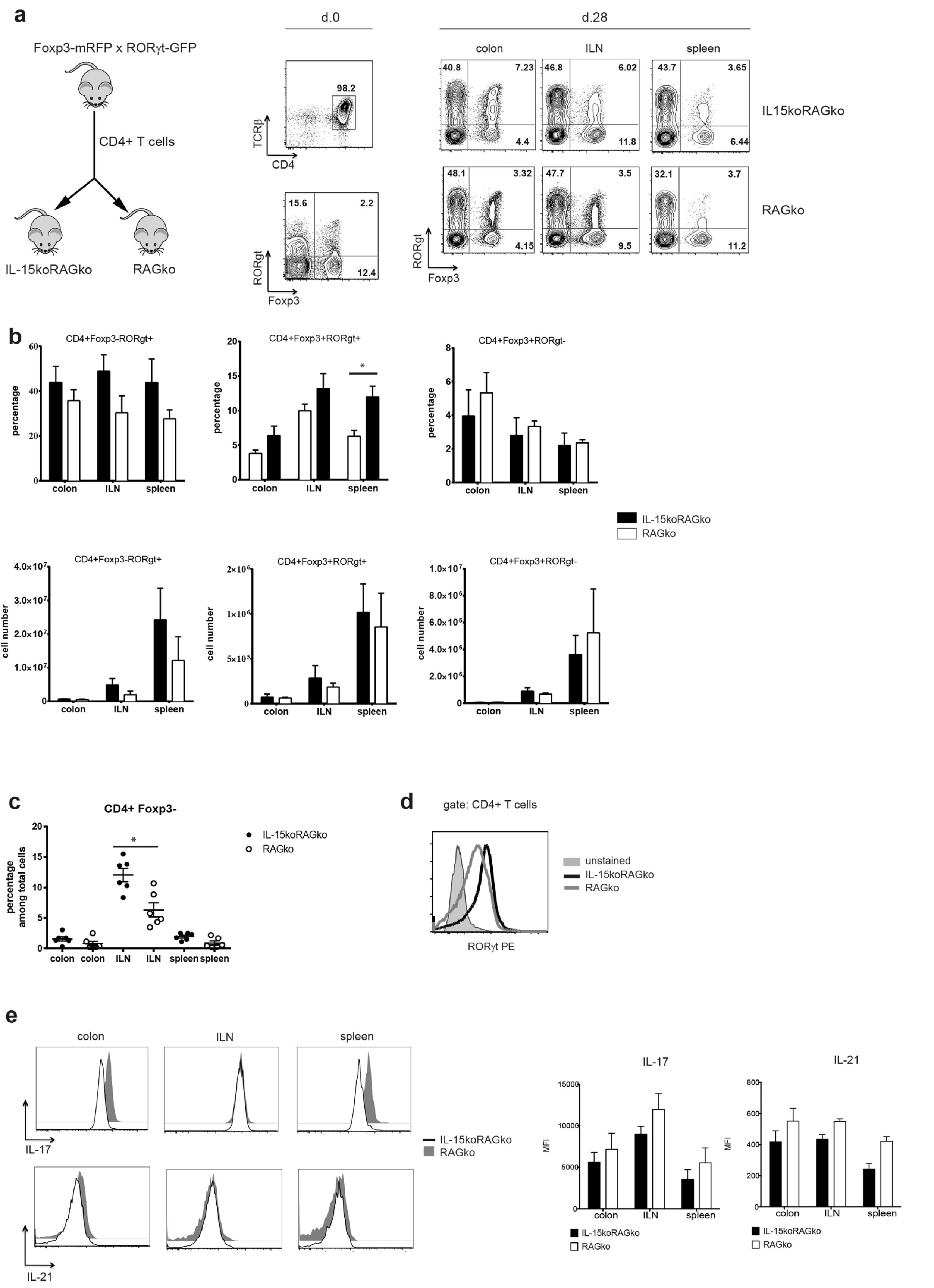

Supplementary Figure 1: CD4+ T cells are plastic in immunodeficient hosts (a) $2x10^5$ CD4+ T lymphocytes purified from double-reporter Foxp3-mRFP x RORγt-GFP mice were transferred into RAGko or IL-15koRAGko mice, which were sacrificed 28 days later: purity of CD4+ T cells (upper panel) and Foxp3 (mRFP) and RORγt (GFP) expression by CD4+ T cells before (day 0, lower panel) and after transfer in the colon, inguinal lymph nodes (ILN) and spleen of injected IL-15koRAGko and RAGko mice (right panel). (b) Percentage among live CD4+ T cells (upper panel) and number (lower panel) of three CD4+ T cell populations: RORγt-Foxp3+, RORγt+Foxp3+, RORγt+Foxp3- in the colon, ILN and spleen of IL-15koRAGko and RAGko recipients 4 weeks after CD4+ T cell transfer. (c) $2x10^5$ CD4+Foxp3+ T lymphocytes (Treg) purified from Foxp3-mRFP mice were transferred into RAGko or IL-15koRAGko mice, which were sacrificed 28 days later (like in Fig.1a): frequency of CD4+Foxp3- T cells among all live cells in the colon, ILN and spleen of IL-15koRAGko and RAGko hosts. (d) Anti-RORγt Ab staining of CD4+ T cells recovered from IL-15koRAGko and RAGko mice (with negative control) (e) Expression of IL-17 and IL-21 by CD4+RORγt+ T cells re-isolated from IL-15koRAGko and RAGko mice after CD4+Foxp3+ T cell transfer: histograms (left panel) and MFI values (right panel).
Data represent results from at least two independent experiments with min.7 mice. All graphs indicate mean values, each point represents an individual mouse; error bars denote SEM; *$p<0.05$ (Student's t test).

Supplementary Figure 2

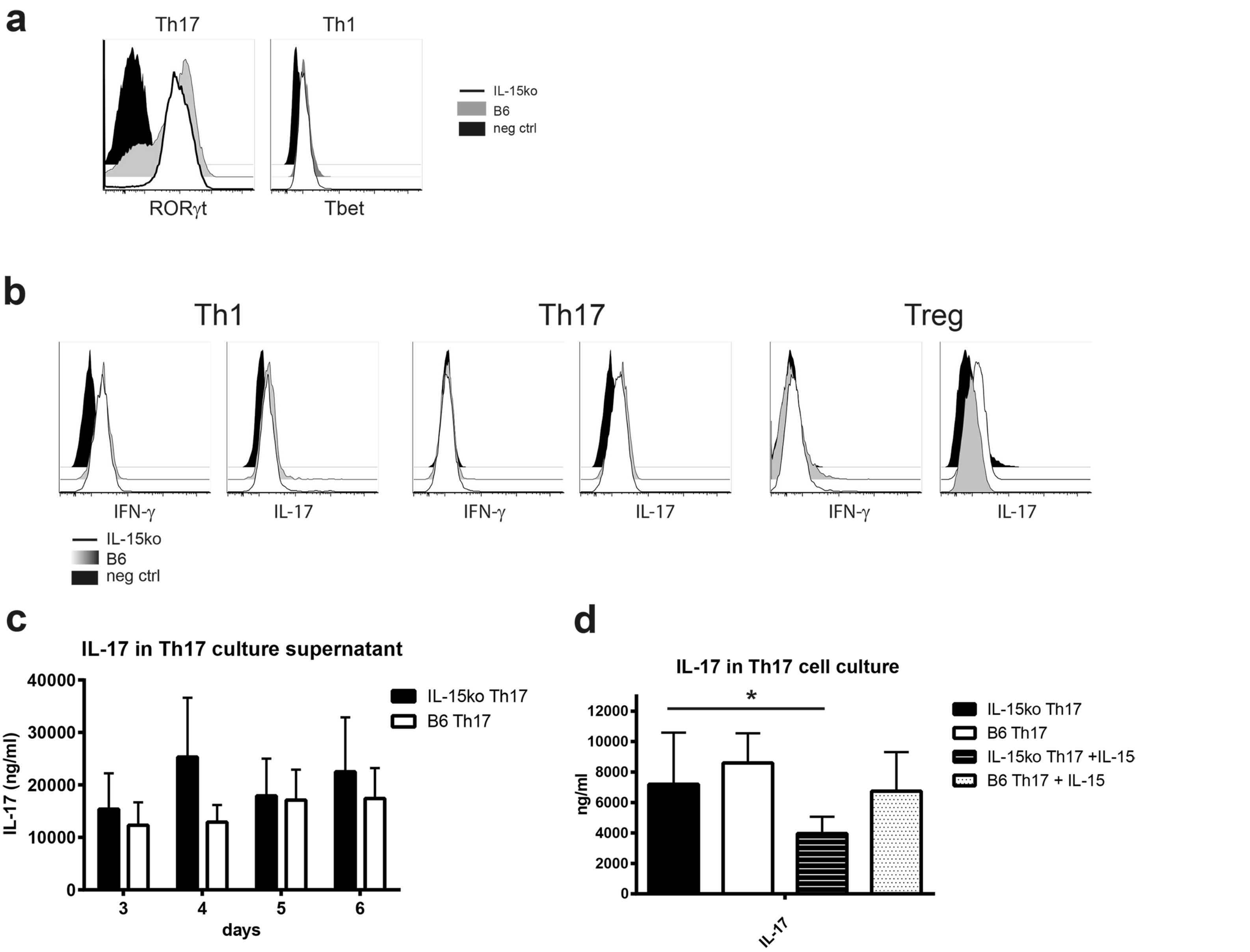

Supplementary Figure 2: IL-15 down-modulates RORγt expression and fine-tunes IL-17 production in vitro (a) Naive CD4+ T lymphocytes from IL-15ko (black line) or B6 mice (grey shadow) were cultured 5 days in vitro under Th17 or Th1 - polarizing conditions. Histograms show RORγt expression in Th17 cells (left) and Tbet in Th1 cells (right) as compared to unstained negative control. (b)Intracellular staining of IFN-γ and IL-17 in B6 and IL-15ko CD4+ T cells cultured in vitro under Th1, Th17 and Treg – differentiating conditions. Black lines: IL-15ko, grey shadows: B6, black shadows: unstained control. (c) Kinetics of IL-17 production in cell culture supernatants: naive CD4+ T lymphocytes from IL-15ko and B6 mice were cultured in Th17-differentiating conditions during 6 days. Concentration of IL-17 was measured by ELISA in cell culture supernatants. (d) Naive CD4+ T lymphocytes from IL-15ko and B6 mice were cultured like in (c) in presence or absence of recombinant IL-15: concentration of IL-17 measured by ELISA in cell culture supernatants. Data represent results from at least two independent experiments with min.6 mice. All graphs indicate mean values, error bars denote SEM; *p<0.05 (Student's t test).

Supplementary Figure 3

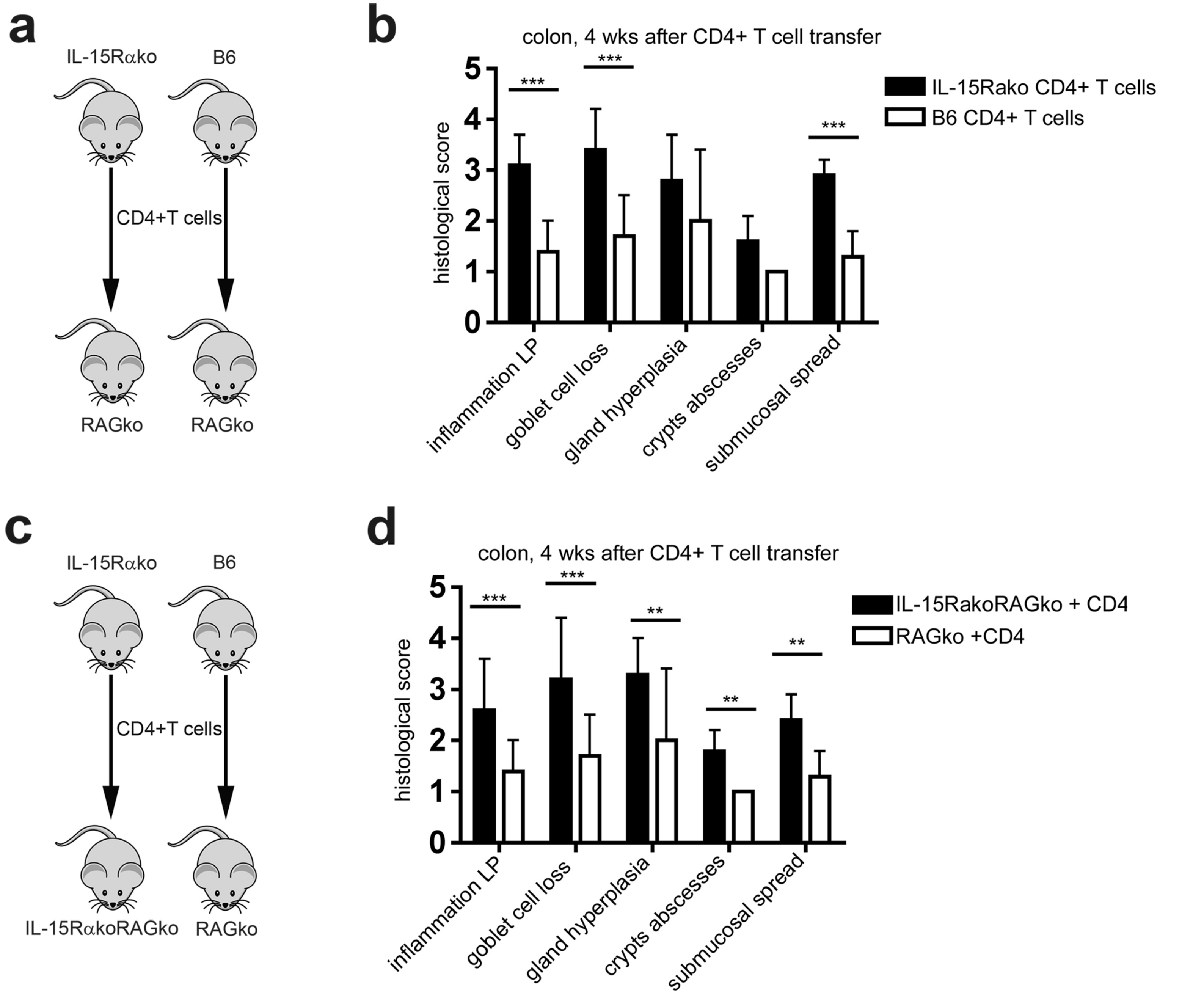

Supplementary Figure 3: Impaired delivery of IL-15 to CD4+ T cells results in a prominent damage to intestinal mucosa.

(a) CD4+ T cells purified from IL-15-receptor alpha deficient (IL-15Rαko) or WT B6 mice were transferred into RAGko mice, which were sacrificed 4 weeks later. (b) Histological scores (from 0: minimal to 5: severe) of microscopic changes in the colon of mice following CD4+ T cell transfer. White bars: RAGko mice injected with B6 CD4+ T cells, black bars: RAGko mice injected with CD4+ T cells from IL-15Rαko mice.

(c) Left panel: CD4+ T cells purified from IL-15Rαko mice were adoptively transferred into IL-15RαkoRAGko mice. Right panel: CD4+ T cells purified from WT B6 mice were adoptively transferred into RAGko mice. All recipient mice were sacrificed four weeks later.

(d) Histological scores of microscopic changes in the colon of mice after CD4+ T cell transfer. White bars: RAGko mice injected with B6 CD4+ T cells, black bars: IL-15RαkoRAGko mice injected with CD4+ T cells from IL-15Rαko mice. (b) and (d): cumulative data from two independent experiments with at least three mice per group. Error bar: SEM; unpaired Student's t-test values: **: $0.001<p<0.05$, ***: $p<0.001$.

Supplementary Figure 4

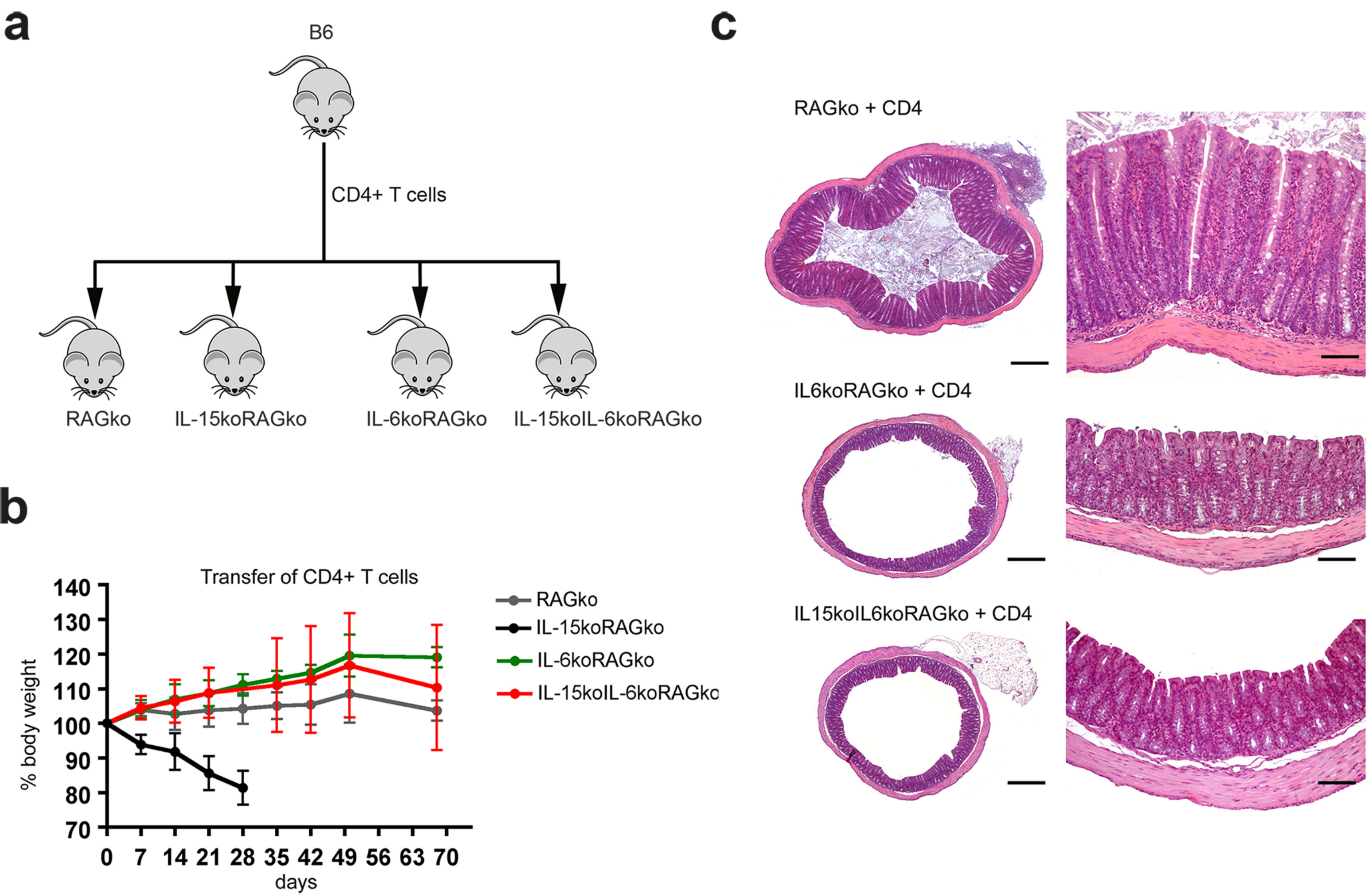

Supplementary Figure 4: Predisposition of IL-15koRAGko mice to CD4+ T cell-mediated premature colitis is prevented in the absence of IL-6. (a) CD4+ T lymphocytes purified from WT B6 mice were transferred into RAGko, IL-15koRAGko, IL-6koRAGko and IL-15koIL-6koRAGko mice, which were euthanized 9 weeks later, except from IL-15koRAGko mice euthanized 4 weeks post transfer due to the rapid body weight loss. (b) Body weight curve (shown as mean percentage of the initial body weight ± standard deviation) measured in RAGko (grey line), IL-15koRAGko (black line), IL-6koRAGko (green line) and IL-15koIL-6koRAGko mice (red line) after CD4+ T cell transfer. (c) Hematoxylin-and-eosin (H&E)-stained microscopic sections of the colon from RAGko (upper panel), IL-6koRAGko (middle panel) and IL-15koIL-6koRAGko mice (lower panel) sampled 9 weeks after CD4+ T cell transfer. Original magnification: left panel-x4, right panel-x10; scale bar-250m. (b)-(c): data from 2 independent experiments with at least 6 mice per group.

Supplementary Figure 5

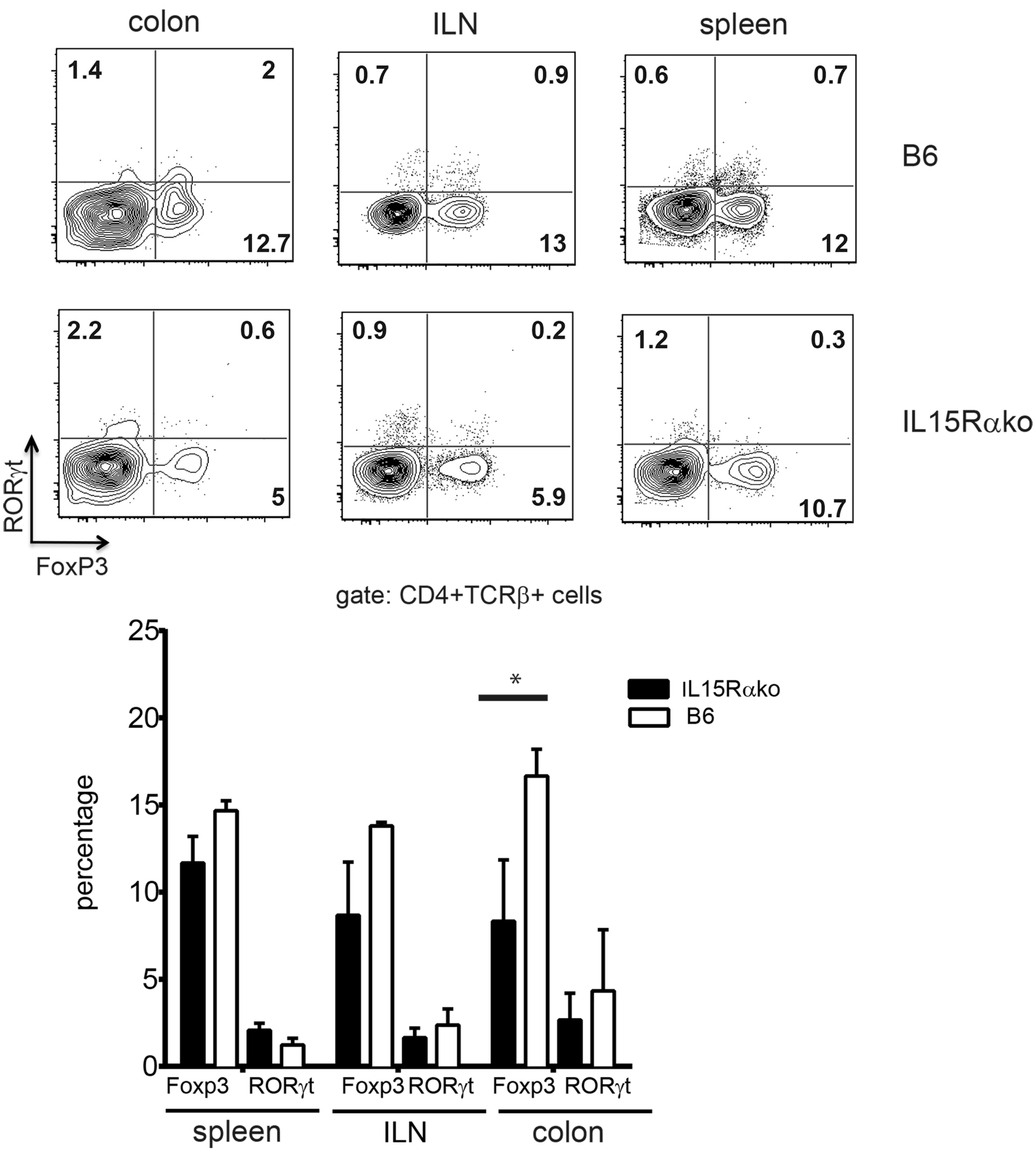

Supplementary Figure 5: Frequency of regulatory T cells decreases significantly in the colon of mice with impaired IL-15 signaling. (a) Expression of RORγt and Foxp3 on CD4+ T cells in the colon, ILN and spleen of unmanipulated IL-15Rαko or B6 mice (b) frequency of RORγt and Foxp3+ cells among CD4+ T cells in the colon, ILN and spleen of unmanipulated IL-15Rαko or B6 mice. (a-b) cumulative data from two independent experiments with at least three mice per group. All graphs indicate mean values; error bars denote SEM *: $p<0.05$ (Student's t-test).

Supplementary Figure 6

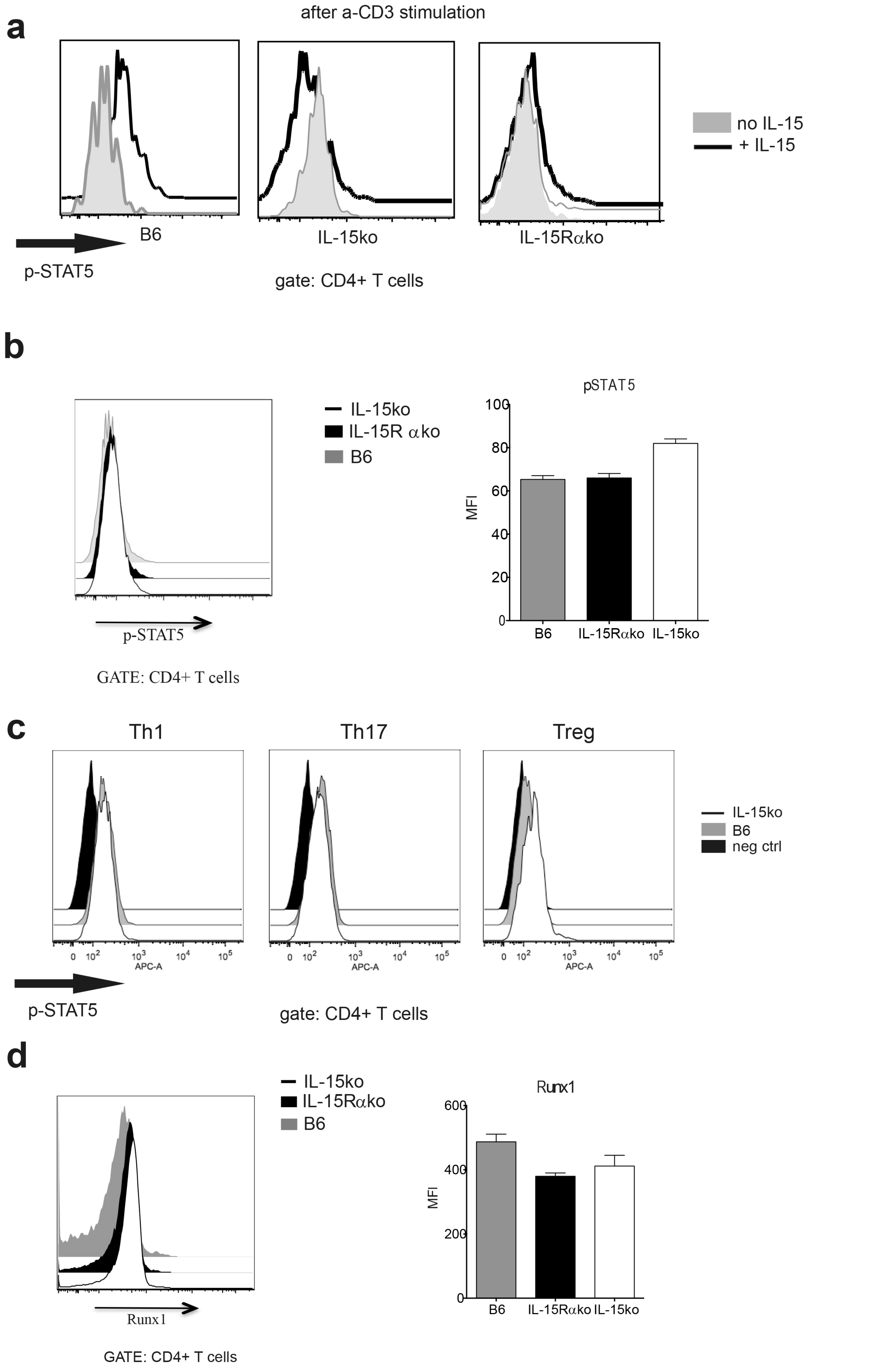

Supplementary Figure 6: Expression of pSTAT5 and Runx1 by IL-15-deficient and B6 CD4+ T cells. (a) IL-15ko, IL-15Rαko (IL-15 receptor alpha deficient) and B6 mice received i.p. 50ug α–CD3 Ab and were sacrificed 2h later. Expression of p-STAT5 (phosphorylated signal transducer and activator of transcription 5) on CD4+ T cells in the LN sampled from the injected mice and stained directly with a-pSTAT5, without restimulation with recombinant mouse (rm) IL-15. (b) Histograms (left) and median fluorescence intensity (MFI) values (right) of p-STAT5 expression on CD4+ T cells isolated from unmanipulated IL-15ko, I L-15Rαko and B6 mice and re-stimulated 20 min in vitro with rmIL-15. (c) Naïve CD4+ T cells from IL-15ko and B6 mice were cultured 5 days in Th1, Th17 and Treg-differentiating conditions (see Suppl. methods for details). Histograms show pSTAT5 expression after 20 min in vitro re-stimulation with rmIL-15. (d) Expression of Runx1 (histograms and MFI values) in CD4+ T cells isolated from LN of unmanipulated IL-15ko, IL-15Rαko and B6 mice. Black line: IL-15ko, black shadow: IL-15Rαko, grey shadow: B6. (a-d) Cumulative data from two independent experiments with at least three mice per group. All graphs indicate mean values; error bars denote SEM.

Supplementary Figure 7

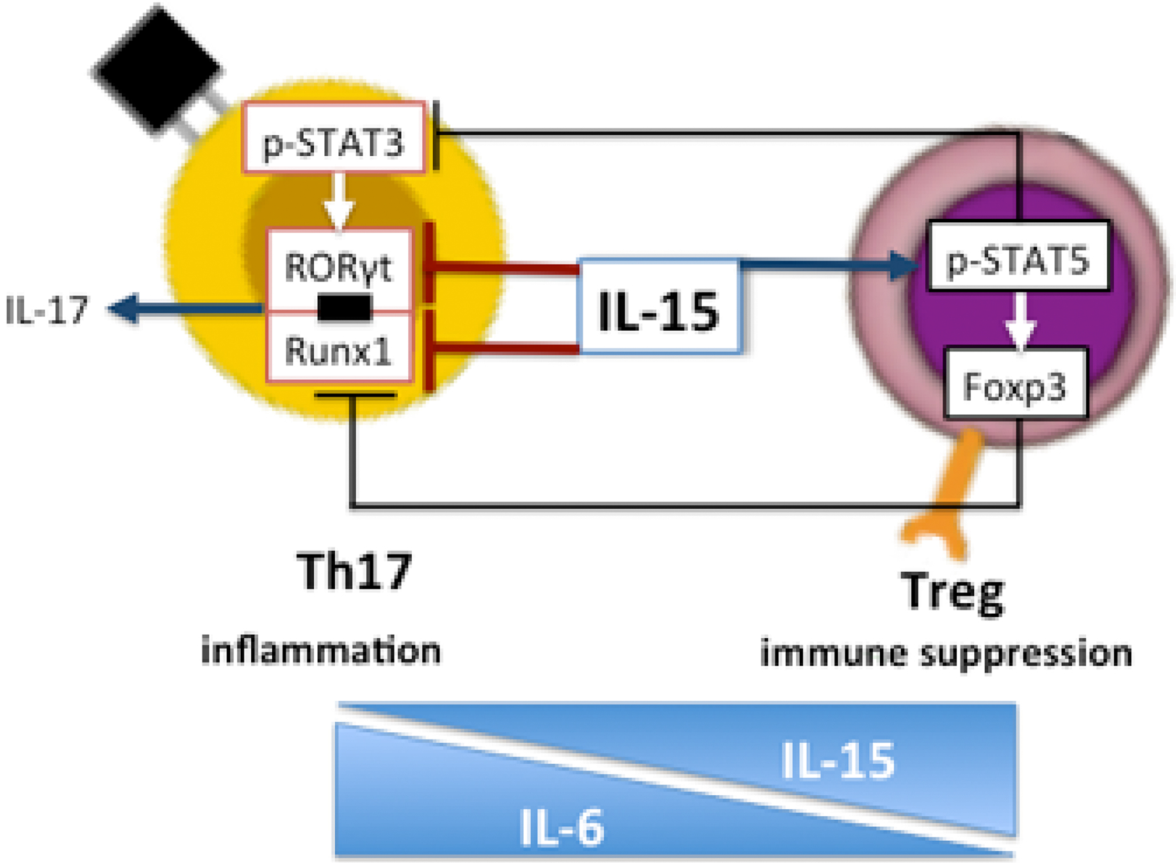

Supplementary Figure 7: Proposed model of IL-15-mediated CD4+ T cell homeostasis. Presence of IL-15 in the local cytokine milieu may shift the CD4+ T cell differentiation towards up-regulation of Foxp3 through the signaling down-stream of p-STAT5, leading in consequence to the acquisition of Treg phenotype. Reversely, low IL-15 availability could promote the induction of RORgt: directly and/or by up-regulation of Runx1 transcription factor, which cooperates with RORgt. Both, RORgt and Runx1 can bind to Il17 locus and thus promote IL-17 expression and induction of Th17 phenotype in CD4+ T cells. IL-6 and IL-15 could play opposite roles in Treg and Th17 differentiation process.

Black lines indicate previously known regulatory pathways; thick lines and gradients summarize the key findings of this study.
